# Selective attenuation of neural responses to own infant’s face in early motherhood: a longitudinal magnetoencephalography study

**DOI:** 10.1093/scan/nsag073

**Published:** 2026-09-02

**Authors:** Elisa Vuoriainen, Piia Astikainen, Xueqiao Li, Qianru Xu, Santeri Yrttiaho, Mikko J Peltola

**Affiliations:** Human Information Processing Laboratory, Faculty of Social Sciences, Tampere University, Tampere, FI-33014, Finland; Jyväskylä Centre for Interdisciplinary Brain Research, Department of Psychology, University of Jyväskylä, Jyväskylä, FI-40014, Finland; Jyväskylä Centre for Interdisciplinary Brain Research, Department of Psychology, University of Jyväskylä, Jyväskylä, FI-40014, Finland; Jyväskylä Centre for Interdisciplinary Brain Research, Department of Psychology, University of Jyväskylä, Jyväskylä, FI-40014, Finland; Center for Machine Vision and Signal Analysis, University of Oulu, Oulu, FI-90014, Finland; Human Information Processing Laboratory, Faculty of Social Sciences, Tampere University, Tampere, FI-33014, Finland; Human Information Processing Laboratory, Faculty of Social Sciences, Tampere University, Tampere, FI-33014, Finland

**Keywords:** early motherhood, caregiving experience, own infant face, LPP, MEG, attentional responses

## Abstract

The transition to parenthood is associated with heightened neural responsiveness to infant cues, particularly to one’s own infant. Yet it remains unclear how this sensitivity changes with caregiving experience. Magnetoencephalography was recorded from 16 first-time mothers viewing images of their own infant, an unfamiliar infant, and an unfamiliar adult at two time points spanning infancy and early toddlerhood. We examined early orbitofrontal activity (∼140 ms), structural face encoding (M170, ∼170 ms), and later motivated attention indexed by the late positive potential (LPP, ∼300–600 ms). Source-level brain data were analyzed with a depth-weighted minimum-norm estimate. Results showed that early LPP responses in the fusiform cortex and late LPP responses in fusiform and occipital regions were larger for own-infant faces at the 1st session but decreased to the level of other faces by the 2nd session. In contrast, early orbitofrontal activity and M170 responses were not modulated by face type or session, and M170 responses showed high temporal stability. These findings suggest that neural activity associated with motivated attention to one’s own infant attenuates across early motherhood, whereas early responses reflecting perceptual and affective processes remain stable. The findings provide longitudinal evidence for changes in maternal neural reactivity to one’s own infant.

## Introduction

The transition to parenthood involves neurobiological changes that support sensitive caregiving and motivated responding to infant cues (Kim et al. 2010a, 2018, Hoekzema et al. 2017, 2020). These adaptations occur in brain regions involved in motivation, emotion regulation, and social cognition, and have been linked to parenting behaviors such as maternal sensitivity (Dufford et al. 2019). Infant faces are particularly salient social stimuli that capture attention and elicit caregiving behavior, partly due to “baby schema” features (Lorenz 1943, Brosch et al. 2007, Glocker et al. 2009, Marsh 2019). Viewing infant faces activates attention- and reward-related neural systems (Glocker et al. 2009, Luo *et al*. 2015) and elicits enhanced attention-related electrophysiological responses (Vuoriainen et al. 2022). Notably, one’s own infant evokes particularly strong neural activation in regions supporting reward, emotion regulation, memory, and mentalizing (Bartels and Zeki 2004, Leibenluft et al. 2004, Rilling 2013, Luo et al. 2015).

Although cross-sectional studies consistently demonstrate heightened neural responsiveness to one’s own infant, little is known about how this sensitivity evolves with caregiving experience. Early postpartum has been described as a period of heightened parental preoccupation and focused attention toward the infant (Winnicott 1975/1956, Leckman et al., 1999, Kim et al. 2013), which has been shown to decline across the first postpartum months (Kim et al. 2013). Structural brain changes during this period in regions implicated in maternal motivation and social information processing support this early window of heightened responsiveness (Kim et al. 2010a, Luders et al. 2020). However, it remains unclear whether enhanced neural sensitivity persists across early motherhood or attenuates as caregiving routines become established.

Electrophysiological methods allow dissociation of temporally distinct perceptual, affective, and attentional stages of face processing, providing a framework for examining how caregiving experience may influence these processes. Early face-sensitive responses include the N170/M170 (∼170 ms), which reflects structural encoding of faces and is typically localized to occipitotemporal regions, with source activity traced to the fusiform (FF) gyrus (Bentin et al. 1996, Bentin and Deouell 2000, Eimer 2000, Gao et al. 2019). Some studies report stronger N170 responses to infant faces in parents than non-parents, suggesting enhanced perceptual encoding of infantile features (for a meta-analysis, see Vuoriainen et al. 2022). Later components such as the P3 and late positive potential (LPP; ∼300–600 ms) index motivated attention toward emotionally salient stimuli (Cuthbert et al. 2000, Polich 2007, Olofsson et al. 2008, Hajcak and Foti 2020), and several studies report enhanced P3/LPP responses to one’s own child (Grasso et al. 2009, Bernard et al. 2018, Vuoriainen et al. 2022). In magnetoencephalography (MEG) studies, comparable late responses have been observed in a similar time window, localized at prefrontal and occipitoparietal regions (Moratti et al., 2011). MEG enables source-level separation of these processing stages and allows identification of early orbitofrontal cortex (OFC) activity (∼130–150 ms), interpreted as rapid affective valuation of infant cues (Kringelbach et al. 2008, Parsons et al. 2013a, 2013b, Young et al. 2016).

Parental experience appears to modulate attention-related responses to infant faces, with first-time mothers showing larger P3 responses to unfamiliar infants than multiparous mothers (Maupin et al. 2019, Bunderson et al. 2020). This has been interpreted either as increased engagement with infant-related cues in first-time mothers or, alternatively, as more efficient processing of such cues in experienced mothers (Bunderson et al. 2020). In contrast, N170 responses have not been found to differ by parity (Maupin et al. 2019), consistent with the view that the N170 reflects relatively automatic structural encoding of faces (Bentin and Deouell 2000, Eimer 2000). However, to our knowledge only two longitudinal studies have examined electrophysiological responses to infant faces during the postpartum period (Bick et al. 2013, Bunderson et al. 2020), and neither investigated biological mothers’ responses to their own infant.

The present longitudinal MEG study addressed this gap by examining neural responses to own-infant, unfamiliar-infant, and adult faces in first-time mothers at two time points spanning 5–16 months postpartum. We investigated early OFC activity (∼140 ms), structural face encoding indexed by the M170 component, and motivated attention reflected in a late response occurring around 300–600 ms. For clarity, we refer to this later response as the LPP throughout the remainder of the text.

We hypothesized enhanced OFC and LPP responses to the own infant compared to unfamiliar infant and adult faces at both assessments, reflecting greater attentional engagement with one’s own infant. We further predicted that attentional engagement would decline with increasing caregiving experience, resulting in attenuation of attention-related responses (LPP), and possibly also early affective responses (OFC), from the first to the second assessment, whereas the M170 would remain stable. This reduction was expected to be most pronounced for responses to the own infant’s face, although a weaker attenuation might also be observed for unfamiliar infant faces.

## Methods

### Participants

A total of 24 first-time mothers were recruited for the study from the Jyväskylä region using population registry information. Twenty participated in the first MEG session, and sixteen returned for a follow-up session approximately 6 months later. The final sample reported here consists of the 16 participants for whom data from both sessions were available.

A power calculation was used to guide the recruitment planning. An a priori power analysis was conducted in G*Power using *F* tests, repeated-measures ANOVA, and a within factors design (Faul et al. 2007). The calculation was intended to guide recruitment for the primary within-subject Face Type × Session repeated-measures design. Because no directly comparable longitudinal study was available, the effect-size assumption was informed by previous ERP studies reporting own-child effects in P3/LPP (e.g. Grasso et al. 2009, Bick et al. 2013, Bernard et al. 2018, Kuzava and Bernard 2018). Together, these studies demonstrated robust own-child effects across infancy and early childhood, supporting the expectation that an own-child response advantage would be detectable at both assessments in the present study. However, they did not provide an empirical estimate of the longitudinal attenuation of this effect. Because the reported cross-sectional effects were generally large, a medium effect size (*f* = .25, Cohen 1988) was adopted as a conservative planning assumption to account for potential attenuation across sessions. Assuming α = .05, power = .80, one group, six repeated measurements (three stimulus categories assessed at two sessions), an assumed correlation of .50 among repeated measurements, and ε = 1, the required sample size was estimated to be *N *= 19. Recruitment was constrained by the availability of eligible first-time mothers and participant retention across the longitudinal study.

Inclusion criteria required that the infant was the mother’s first child, born full-term (≥37 weeks), and without major facial anomalies (detailed recruitment process and inclusion and exclusion criteria are provided in the Supplementary Materials). Mothers were right-handed, had normal or corrected-to-normal vision, and reported no major neurological or psychiatric conditions. All procedures were approved by the Ethics Committee of the Tampere Region and were conducted in accordance with the Declaration of Helsinki. The participants provided written informed consent.

The mean age of the participants was 31.9 years (SD = 3.5, range 26–38 years). The majority had a higher education degree (either University degree or University of Applied Sciences). At the first visit all the mothers were on parental leave, whereas at the second visit 10 out of 16 mothers had returned to work or studies.

### Stimuli

The stimuli consisted of three face-image categories: (1) the participant’s own infant, (2) an unfamiliar infant, and (3) an unfamiliar adult female.

Mothers provided photographs of their infant with a neutral expression; one image per participant was selected based on clarity and frontal orientation and a relatively neutral facial expression. The selected photograph was used at both measurement sessions to ensure that longitudinal changes could not be attributed to changes in facial appearance, maturity, or other stimulus characteristics over time. Unfamiliar infant faces were drawn from the Tromsø Infant Faces Database (Maack et al. 2017) and supplemented with age- and gender-matched images from other participants when necessary (a written consent for such secondary use of the images was obtained in advance). Adult face images were selected from the NimStim set (Tottenham et al. 2009) and the Karolinska Directed Emotional Faces database (KDEF; Lundqvist et al. 1998).

Unfamiliar infant images were selected to approximate the age and gender of each participant’s own infant. For adult faces, only female identities were used, as all participants were women. Across all stimulus categories, images were selected so that mouth position and overall appearance (e.g. skin tone) were broadly comparable within each participant’s stimulus set. For each participant, one unfamiliar infant and one adult identity were selected from the databases, resulting in variability across participants and minimizing repeated presentation of the same identities. For all external databases, only identities previously validated as displaying neutral facial expressions were included (see Supplementary Materials for details).

**Figure 1:**
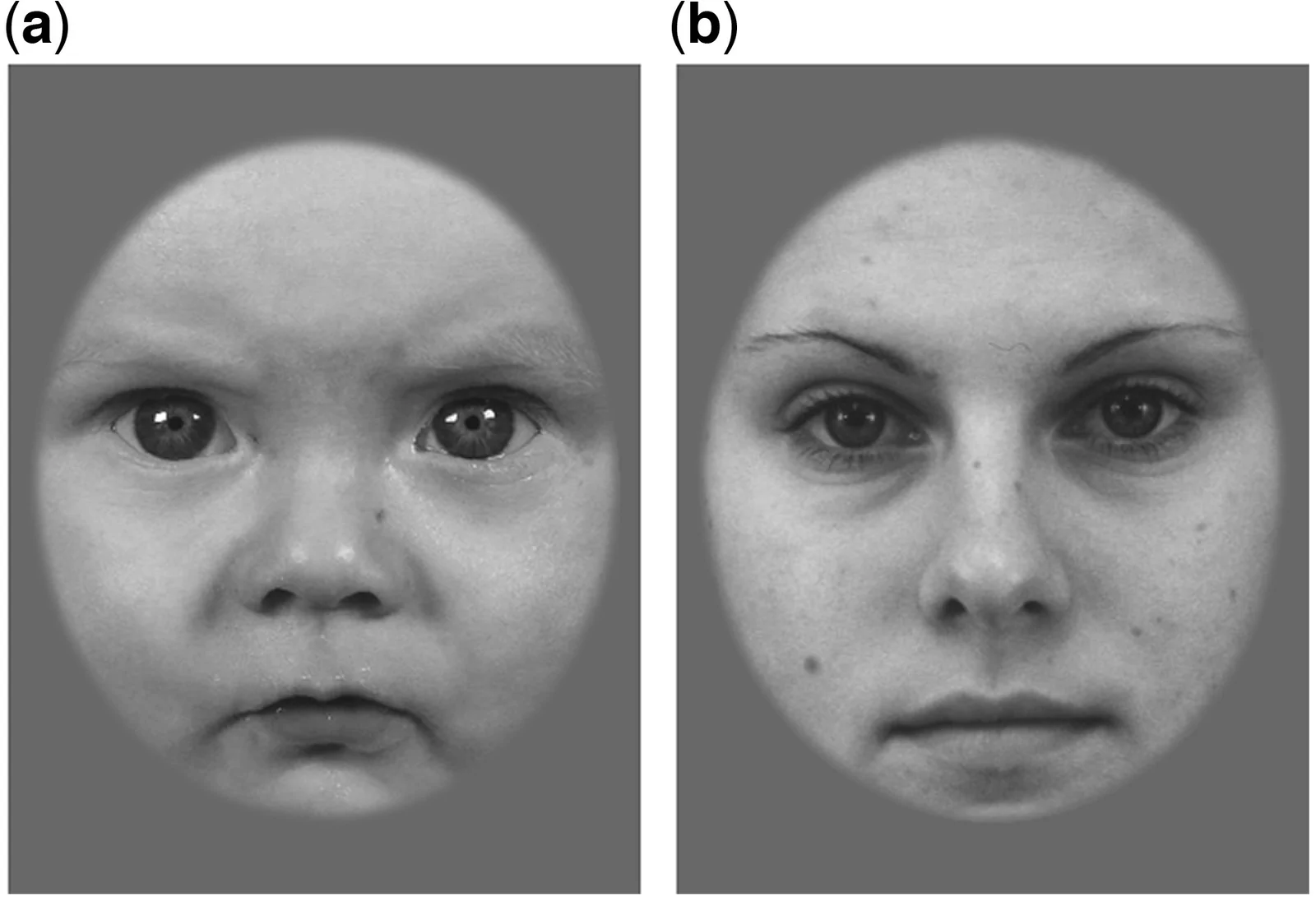
Illustration of the facial stimuli. (a) The infant face (A18M7-7841NE) taken from the Tromsø Infant Faces Database and (b) the adult face (AF26NES) from the Karolinska Directed Emotional Faces represent categories of unfamiliar infant and unfamiliar adult female, respectively. The same oval crop was applied to the image of the participant’s own infant.

All images were converted to grayscale, cropped with an oval mask to remove external features (see Fig. 1), and matched for luminance. The same own-infant, unfamiliar infant, and adult images were used at both sessions.

### Procedure

Each participant completed two identical measurement sessions approximately 6 months apart (mean interval = 26.9 weeks, range 25–29 weeks). The 1st session took place when infants were on average 31.4 weeks old (SD = 7.2, median = 31 weeks, range 20–40 weeks, corresponding to ∼5–9 months). At the 2nd session, infants were on average 58.5 weeks (SD = 7.2, median = 58 weeks, range = 46–68 weeks, corresponding to ∼10–16 months).

Faces were presented in an oval shape against a gray background at a viewing distance of approximately 100 cm (visual angle 4.9° × 5.7°). Each trial consisted of a 1-s face presentation followed by a central fixation cross displayed for a jittered interval of 1–1.5 s. In 10% of trials the fixation cross appeared in red, and participants were instructed to press a button when this occurred.

Images were presented in pseudorandom order with the restriction that the same image was not shown consecutively. The task consisted of two blocks, each containing 50 presentations of each face category, resulting in 100 repetitions of each identity.

After the face task, participants rated the three stimulus images in terms of emotional expression, clarity, and valence. Own-infant faces were rated as more positively valenced than unfamiliar infant and adult faces at both sessions, and no differences between sessions were observed. Detailed results are provided in the Supplementary Materials.

### MEG data acquisition

MEG data were recorded with a 306-channel Elekta Neuromag TRIUX™ system (Elekta AB, Stockholm, Sweden) in a magnetically shielded room. The data were sampled at 1000 Hz and filtered online with a 0.1–330 Hz band-pass filter.

Head position was continuously tracked using five HPI coils. Structural T1- and T2- weighted MRI scans were acquired for each participant to enable source modeling at a private clinic (Synlab, Jyväskylä, Finland) using a 1.5 T MRI scanner (Signa HDxt, GE Healthcare, Chicago, IL, USA). Electrooculogram and electrocardiogram were recorded for artifact correction. During the recording the participant was seated in an upright position. For more details on data acquisition, see Supplementary Materials.

### Preprocessing of MEG data

MEG data were preprocessed using MaxFilter 3.0 (Elekta AB) with spatiotemporal signal space separation (tSSS; 30-s buffer) to suppress external interference and correct for head movements (Taulu and Simola 2006). Bad channels were identified and reconstructed.

Structural MRI data (T1 and T2) were processed with FreeSurfer’s (Dale et al. 1999, Fischl et al. 1999; RRID: SCR_001847) *recon-all* pipeline to obtain cortical surfaces for source modeling. Data were further processed in Brainstorm (Tadel et al. 2011), where individual anatomies were co-registered with MEG data using fiducial points (manually set) and a digitized head shape. The registration was visually inspected, and the sensor array was manually adjusted when necessary to improve alignment with the head surface.

Continuous data were low-pass filtered at 40 Hz. Cardiac and ocular artifacts were corrected using signal-space projection (SSP), and remaining contaminated segments were excluded based on visual inspection. When no clear SSP components were identified for cardiac or ocular artifacts, cardiac correction was omitted, and trials with large eye-movement activity (EOG > 200 μV) were excluded. Data were epoched from −100 to 800 ms relative to stimulus onset and baseline-corrected using the pre-stimulus interval. The trials immediately following a button press were excluded to disregard data contaminated with motor responses. Epochs were averaged separately for each face type.

Source reconstruction was performed using a depth-weighted minimum-norm estimate (wMNE; Hämäläinen and Ilmoniemi 1994, Lin et al. 2006) with unconstrained source orientation, and source amplitude was defined as the norm of the three dipole components at each vertex. Both magnetometer and gradiometer data were included in the noise covariance estimation and source reconstruction. Noise covariance was estimated from the pre-stimulus baseline (−100 to 0 ms) calculated over all conditions. Source estimates were normalized using dynamic statistical parametric mapping (dSPM), resulting in unitless, noise-normalized values.

Regions of interest (ROIs) were defined using the Desikan–Killiany atlas (Desikan et al. 2006) and based on prior literature and inspection of the grand-average source maps. Early OFC activity (∼100–230 ms) was extracted from the right lateral and medial OFC (Kringelbach et al. 2008, Parsons et al. 2013a, 2013b). The M170 (∼100–230 ms) was quantified from the right FF gyrus (Gao et al. 2019, Bentin and Deouell 2000). For the LPP (250–400 ms and 400–600 ms), activity was extracted from the right FF, lingual, and lateral occipital cortices (Moratti et al. 2011, Liu et al. 2012). Peak amplitude was used for the M170, whereas mean activity within the respective time windows was used for the OFC and LPP.

### Statistical analysis

Statistical analyses were conducted in IBM SPSS Statistics (version 32.0.0), except for exploratory infant-age analyses, which were conducted in R (version 4.5.1).

Repeated measures analysis of variances (ANOVAs) were conducted separately for each component with the within-subject factors Face Type (own infant, unfamiliar infant, adult), Session (1st, 2nd session), and ROI (defined per component).

When a significant Face Type × Session × ROI interaction emerged, follow-up ANOVAs were run within each ROI. If the three-way interaction was absent but a significant Face Type × Session interaction was present, responses were averaged across ROIs. Significant main effects of Face Type were examined with Bonferroni-corrected pairwise comparisons. Significant Face Type × Session interactions were followed with two-sided *t*-tests comparing sessions within each face type and face types within each session. The follow-up comparisons were corrected for multiple testing using the false discovery rate (FDR; Benjamini and Hochberg 1995).

To assess stability across time, Pearson correlations were computed between sessions for each face type and component, with FDR correction applied to control for multiple comparisons.

To examine whether variability in infant age at study entry influenced the observed longitudinal response patterns, additional exploratory analyses were conducted. Linear mixed-effects models including Face Type, Session, Infant Age at Session 1, and their interactions were fitted separately for each neural outcome measure. These comprised the M170 response, the early OFC response (with medial and lateral OFC combined because no ROI effects were observed in the primary analyses), and the early and late LPP responses in the FF, occipital, and lingual regions. Participants were included as a random intercept. In addition, exploratory Pearson correlations were conducted to examine associations between infant age and the magnitude of the own-infant bias (own minus unfamiliar infant responses) at each assessment, using the corresponding infant age at each session. Associations between infant age at Session 1 and longitudinal changes in responses to one’s own infant face were also examined.

## Results

**Figure 2:**
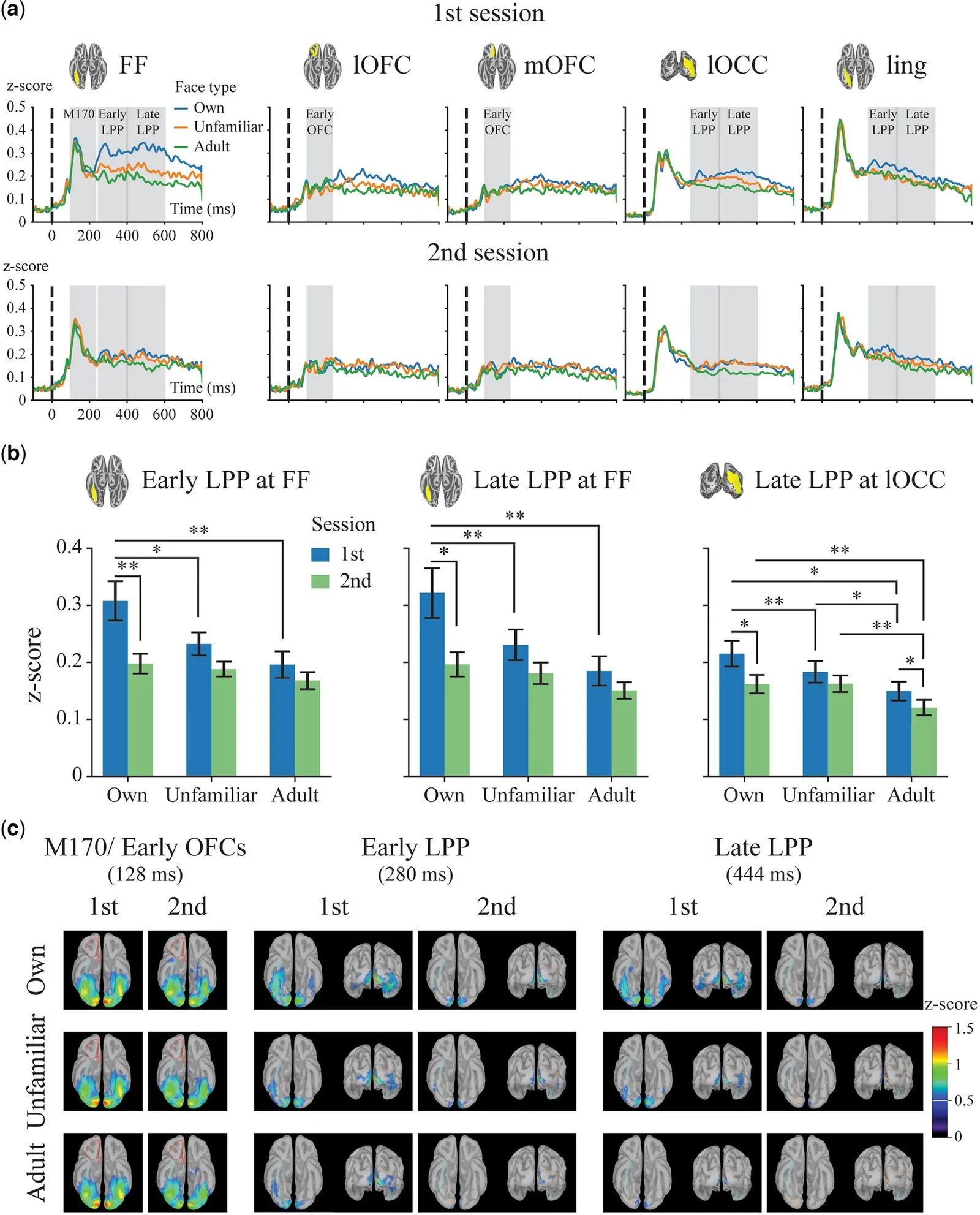
(a) Source activity waveforms for each Face Type and ROI in the 1st and 2nd sessions. Gray shading marks the time windows of each MEG component. The topography maps indicate the selected ROI (highlighted in yellow). The maps show inferior and posterior views. (b) Mean amplitude values for each Face Type in MEG components showing significant pairwise differences either between Face Types within a session or for the same Face Type across sessions. Asterisks indicate significant differences (*P < .05, **P < .01, ***P < .001). (c) Source activity topography maps for each ROI and MEG component for each Face Type and session. Maps are displayed using amplitudes standardized with Brainstorm’s dynamic statistical parametric mapping (dSPM) method, resulting in values expressed as z-scores. The activity is shown at the peak timepoint indicated in parentheses below each component name. Session labels “1st” and “2nd” refer to the first and second measurement sessions, respectively. For the M170 and early OFC responses, topographies are shown from the inferior view to highlight activity in the fusiform (FF) and orbitofrontal (OFC) regions. For the LPP, both inferior and posterior views are presented to illustrate activity in the fusiform (FF), lateral occipital cortex (lOCC), and lingual gyrus (Ling).

Source activity waveforms and topographies for each Face Type and ROI are shown in Fig. 2 and mean (SD) dSPM-normalized source activity for ROIs showing significant Face Type × Session interactions are presented in Table 1.

**Table 1:** Mean (SD) responses for each Face Type and Session in ROIs showing significant interactions.

Component	ROI	Session	Own	Unfamiliar	Adult
Early LPP	FF	1st	0.31 (0.14)	0.23 (0.08)	0.20 (0.09)
Early LPP	FF	2nd	0.20 (0.07)	0.19 (0.05)	0.17 (0.06)
Late LPP	FF	1st	0.32 (0.17)	0.23 (0.11)	0.18 (0.10)
Late LPP	FF	2nd	0.20 (0.09)	0.18 (0.08)	0.15 (0.06)
Late LPP	lOCC	1st	0.22 (0.09)	0.18 (0.08)	0.15 (0.07)
Late LPP	lOCC	2nd	0.16 (0.06)	0.16 (0.06)	0.12 (0.05)

*Note*. Values represent mean (SD) dSPM-normalized source activity (unitless).

### Early OFC

For early OFC a repeated-measures ANOVA revealed no significant Face Type × Session × ROI interaction, but a significant Face Type × Session interaction, *F*(2, 30) = 4.65, *P* = .022, η_p_^2^= .24. Therefore, responses were averaged across ROIs (mOFC, lOFC) for each Face Type within each session and used in pairwise comparisons to follow up the interaction.

For the Face Type × Session interaction, follow-up comparisons indicated no significant differences in responses to face types within or between sessions.

### M170

A repeated-measures ANOVA with Face Type (own infant, unfamiliar infant, adult), and Session (1st, 2nd) was conducted for the right FF region. Neither the main effect of Face Type nor the Face Type × Session interaction was significant.

### Early LPP

At 250-400 ms, a repeated measures ANOVA showed a significant Face Type × Session × ROI interaction effect, *F*(4, 60) = 2.96, *P* = .027, η_p_^2^ = .17. Therefore, analyses were conducted separately for each ROI.

For FF region there was a significant main effect of Face Type, *F*(2, 30) = 9.79, *P* < .001, η_p_^2^ = 0.40, and a Face Type × Session interaction, *F*(2, 30) = 8.66, *P* = .001, η_p_^2^ = 0.37.

For Face Type main effect, the follow-up pairwise comparisons showed larger responses to own infant face, (*M *= 0.25, SD = 0.09), than unfamiliar infant face, (*M *= 0.21, SD = 0.05), *P* = .046, Cohen’s *d *= 0.55, and compared to adult face, (*M *= 0.18, SD = 0.06), Cohen’s *d *= 0.92, *P* = .003, across sessions.

For the Face Type × Session interaction, follow-up comparisons indicated that responses to the own-infant face decreased from the 1st to the 2nd session, *t*(15) = 3.1, *P* = .009, Cohen’s *d *= 0.99, whereas no changes were observed for the other face types. At the 1st session, responses to the own infant face were larger than the unfamiliar infant face, *t*(15) = 3.4, *P* = .015, Cohen’s *d *= 0.70 and the adult face, *t*(15) = 4.4, *P* = .005, Cohen’s *d *= 0.93, whereas no differences were observed for other face types or at the 2nd session.

For the lateral occipital region, there was a significant main effect of Face Type, *F*(2, 30) = 5.3, *P* = .021, η_p_^2^ = 0.26, but pairwise comparisons revealed no significant differences between face types. The Face Type × Session interaction was non-significant.

For the lingual region, there was a significant main effect of Face Type, *F*(2, 30) = 8.27, *P* = .001, η_p_^2^ = 0.36. The Face Type × Session interaction was non-significant.

Follow-up pairwise comparisons showed larger responses for own infant face (*M *= 0.23, SD = 0.06), compared to unfamiliar infant face (*M *= 0.20, SD = 0.06), *P* = .018, Cohen’s *d *= 0.50 and adult face (*M *= 0.20, SD = 0.05), *P* = .003, Cohen’s *d *= 0.54 across sessions.

### Late LPP

At 400–600 ms, a repeated-measures ANOVA, showed a significant Face Type × Session × ROI interaction effect, *F*(4, 60) = 3.62, *P* = .021, η_p_^2^ = .20. Therefore, separate ANOVAs were conducted for each ROI.

For FF region, there was a significant main effect of Face Type, *F*(2, 30) = 14.34, *P* < .001, η_p_^2^ = .49 and an interaction effect of Face Type × Session, *F*(2, 30) = 6.18, *P* = .006, η_p_^2^ = .29.

For Face Type main effect, the follow-up pairwise comparisons, showed larger responses for own infant face (*M *= 0.26, SD = 0.11), compared to unfamiliar infant face (*M *= 0.21, SD = 0.07), *P* = .014, Cohen’s *d *= 0.54, and compared to adult face (*M *= 0.17, SD = 0.07), *P* = .002, Cohen’s *d *= 0.98, and larger responses for unfamiliar infant face compared to adult face, *P* = .027, Cohen’s *d *= 0.57, across sessions.

For the Face Type × Session interaction, follow-up comparisons indicated that responses to own infant face decreased from the 1st to the 2nd session, *t*(15) = 3.1, *P* = .024, Cohen’s *d *= 0.88, whereas no changes were observed for other face types. At the 1st session, responses to own infant face were larger than unfamiliar infant face, *t*(15) = 3.7, *P* = .009, Cohen’s *d *= 0.63 and adult face, *t*(15) = 4.2, *P* = .007, Cohen’s *d *= 1.00. All other comparisons were non-significant.

For the lateral occipital region, there was a significant main effect of Face Type, *F*(2, 30) = 12.50, *P* = .001, η_p_^2^ = .46 and an interaction effect of Face Type × Session, *F*(2, 30) = 3.35, *P* = .049, η_p_^2^ = .18.

For Face Type main effect, the follow-up pairwise comparisons, showed larger responses for own infant face (*M *= 0.19, SD = 0.07), compared to adult face (*M *= 0.14, SD = 0.06), *P* = .007, Cohen’s *d *= 0.77, and larger responses to unfamiliar infant face (*M *= 0.17, SD = 0.07) compared adult face, *P* = .006, Cohen’s *d *= 0.46, across sessions. There was no significant difference between responses to own infant face compared to unfamiliar infant face.

For the Face Type × Session interaction, follow-up comparisons indicated that responses to own infant face decreased from the 1st to the 2nd session, *t*(15) = 2.6, *P* = .034, Cohen’s *d *= 0.78, and responses to adult face decreased from the 1st to the 2nd session, *t*(15) = 2.4, *P* = .034, Cohen’s *d *= 0.49 whereas no changes were observed for unfamiliar infant face. At the 1st session, responses to own infant face were larger than unfamiliar infant face, *t*(15) = 3.6, *P* = .009, Cohen’s *d *= 0.47 and adult face, *t*(15) = 3.3, *P* = .011, Cohen’s *d *= 0.87, and responses to unfamiliar infant face were larger than to adult face, *t*(15) = 2.5, *P* = .037, Cohen’s *d *= 0.40. At the 2nd session, the responses to own infant face were larger than to adult face, *t*(15) = 3.5, *P* = .009, Cohen’s *d *= 0.72, and responses to unfamiliar infant face were larger than to adult face, *t*(15) = 4.33, *P* = .005, Cohen’s *d *= 0.72. Responses to own infant face did not differ from responses to unfamiliar infant face.

For the lingual region, there was a significant main effect of Face Type, *F*(2, 30) = 5.87, *P* = .007, η_p_^2^ = .28. The Face Type × Session interaction was non-significant.

The follow-up pairwise comparisons showed larger responses for own infant face (*M *= 0.19, SD = 0.07), compared to adult face (*M *= 0.16, SD = 0.06), *P* = .026, Cohen’s *d *= 0.46. There was no significant difference between responses to own infant face compared to unfamiliar infant face (*M *= 0.17, SD = 0.05), or unfamiliar infant face compared to adult face.

### Infant age and longitudinal changes in neural responses

Exploratory analyses were conducted to examine whether infant age at study entry was associated with the observed neural response patterns. Neither the Session × Infant Age interaction nor the Face Type × Session × Infant Age interaction was significant for any neural outcome measure (all ps > .16; Table S3).

To further examine associations between infant age and the magnitude of the own-infant bias, correlations were conducted between infant age and the difference in neural responses to own versus unfamiliar infant faces at each session across all components and regions. For these analyses, infant age was defined as the infant’s age at the corresponding measurement session. No correlations reached statistical significance. The strongest associations were observed for the late LPP in the FF and lingual regions at Session 1 (*r *= −.44, *P* = .088, and *r *= −.44, *P* = .092, respectively), whereas all other correlations were non-significant (all *p*s > .18). In addition, infant age at Session 1 was not significantly associated with longitudinal changes in responses to own infant face (all *p*s > .18).

### Correlations across assessment sessions

Correlations between the 1st and 2nd sessions were examined for each face type, component, and ROI.

M170 responses showed positive correlations between the 1st and 2nd sessions for all face types (own infant: *r* = .805, *P* = .002; unfamiliar infant: *r* = .845, *P* = .001; adult: *r* = .910, *P* < .001). Significant correlations were also observed for adult faces in the lateral occipital region for early LPP (*r* = .695, *P* = .016) and late LPP (*r* = .699, *P* = .016). All other FDR-corrected *P*-values were non-significant. Scatterplots of significant correlations are shown in Figure S1.

As an exploratory analysis, correlations between valence ratings of the own-infant face and LPP amplitudes at the 1st session were examined (separately for each ROI and time window). This was done to assess whether the own-infant LPP enhancement observed at the 1st session was associated with subjective evaluation of the stimulus. No significant associations were found.

## Discussion

The present longitudinal MEG study examined how neural responses to one’s own infant evolve across early motherhood by assessing early OFC activity, the M170, and later LPP responses at two timepoints spanning infancy and early toddlerhood. The key finding was a selective attenuation of LPP responses to the mother’s own infant face in FF and occipital regions, whereas early OFC activity and the M170 remained stable across sessions and did not differentiate between face types. Importantly, this attenuation was stimulus-specific: neural responses to unfamiliar infant and adult faces remained largely stable. These results provide novel evidence that motivated attentional responses to one’s own infant decrease as caregiving experience accumulates, while early responses reflecting perceptual and affective processes remain stable.

The own-infant advantage emerged in the LPP time window, a component linked to motivated attention toward emotionally salient stimuli (Olofsson et al. 2008, Ferrey et al. 2016, Hajcak and Foti 2020). The transition to parenthood has been proposed to increase the incentive value of infant cues, resulting in enhanced attentional engagement toward infant-related stimuli (Ferrey et al. 2016). Consistent with this account, parents typically show larger LPP responses to infant faces than non-parents, and enhanced LPP amplitudes to infant faces have been associated with caregiving-related characteristics such as maternal sensitivity (Bernard et al. 2018, Vuoriainen et al. 2022). The robust own-infant LPP advantage observed during the first postpartum year in the present study is consistent with this motivated-attention account. Our longitudinal findings further showed that the magnitude of this attentional advantage decreased as mothers transitioned from parenting an infant to parenting a toddler. Thus, heightened attentional engagement with one’s own infant may change over the course of early parenthood.

The temporal change observed in the present study is partly consistent with but also differs from previous ERP studies examining attention-related responses to infant faces. The few longitudinal studies have primarily examined changes relatively early in the postpartum period. Bick et al. (2013) reported enhanced P3 responses in mothers to foster infants compared with unfamiliar infants across two sessions with the first assessment occurring within the first 2 months of foster placement and the second approximately three months later. Bunderson et al. (2020) observed stable P300 responses to unfamiliar infant faces in mothers between 2 and 7 months postpartum. In line with the latter finding, neural responses to unfamiliar infant faces did not show longitudinal modulation in the present study. Instead, the observed change was specific to responses to one’s own infant. Previous cross-sectional ERP studies have consistently reported enhanced P3/LPP responses to one’s own child compared to unfamiliar children across a range of postpartum ages (e.g. Grasso et al., 2009, Bernard et al., 2018, Kuzava and Bernard 2018, Lowell et al. 2023), although most have likewise focused on the first postpartum year. Thus, relatively little is known about how these responses change with more extended parenting experience. Together, these findings suggest that heightened neural sensitivity to one’s own infant may be characteristic of early parenthood, while the present longitudinal results indicate that this sensitivity may diminish as parenthood progresses.

Although the robust own-infant effect during the first postpartum year is consistent with previous research, the longitudinal attenuation should be interpreted with caution. The present design does not allow us to fully disentangle the potential sources of this longitudinal change. The same infant photograph was used at both measurement sessions and therefore more closely matched the child’s current appearance at the first than at the second assessment. This reduced correspondence with the child’s current appearance may have influenced the attentional or motivational response elicited by the photograph and thereby contributed to the observed LPP attenuation. Thus, the present design cannot determine to what extent the observed attenuation reflects this stimulus-related factor versus changes in motivational engagement across early parenthood.

The observed longitudinal change can also be considered in the context of theoretical accounts and empirical evidence emphasizing the role of learning and caregiving experience in the parental brain. The parental brain has been conceptualized as a plastic and experience-dependent system that becomes progressively fine-tuned through interactions with the infant (Feldman 2015). Consistent with this view, experience with infant cues and learning through parent–infant interactions have been proposed to shape parental processing and responsivity to these cues (Parsons et al. 2013a, Ferrey et al. 2016). Longer duration of motherhood has been associated with greater neural responses to infant emotional vocalizations in brain regions involved in emotional processing, suggesting experience-dependent changes in the processing of infant cues during the first postpartum year (Parsons et al. 2017). In addition, studies comparing first-time and multiparous mothers have reported larger attention-related responses (P300/LPP) to infant faces in first-time mothers (Maupin et al. 2019, Bunderson et al. 2020). These differences have been interpreted as reflecting greater salience and attentional engagement with infant cues in less experienced mothers, or alternatively, more efficient processing in more experienced mothers. The present longitudinal findings extend this literature by showing an attenuation of the own-infant LPP advantage within the same first-time mothers across the transition from parenting an infant to parenting a toddler.

The parental preoccupation framework provides one theoretical account for this longitudinal attenuation. Heightened vigilance and preoccupation with infant cues have been proposed to characterize the transition to parenthood (Winnicott 1975, Leckman et al. 1999) and to decline as the postpartum period progresses (Kim et al. 2013). From this perspective, the observed attenuation of the own-infant LPP advantage may reflect reduction of the heightened attentional engagement characteristic of early parenthood as caregiving routines become established. However, the absence of measurements from the earliest postpartum months limits conclusions about when these changes begin and whether even stronger attentional responses occur earlier.

The dissociation between early and late neural responses further clarifies the processing stage at which modulation occurred. The M170 component, associated with structural face encoding, remained stable across sessions and did not differentiate between face types. This is in line with the small number of previous ERP studies which have investigated parents’ responses to their own child versus unfamiliar child face and found no differences at this time window (see Vuoriainen et al. 2022 for a meta-analysis). Early OFC activity did not differ between face types, which contrasts with some earlier studies (e.g. Kringelbach et al. 2008, Parsons et al. 2013b). One possible explanation for this discrepancy is that those studies included non-parent participants, and it is possible that OFC responses are modulated differently in parents versus non-parents, perhaps due to novelty or differences in emotional salience. Another potential explanation relates to the use of neutral facial expressions in the present study, whereas previous studies often employed emotional expressions or more complex infant stimuli (e.g. infant faces with cleft lip).

The source reconstruction indicated that LPP activity originated primarily from visual cortical regions, especially the FF and occipital cortices. This is consistent with prior studies, which have identified both cortical and subcortical contributors to the LPP, including visual areas (Sabatinelli et al. 2007, 2013, Liu et al., 2012). Visual cortex engagement during motivationally salient stimuli has been linked to a subcortical–cortical feedback system, in which subcortical signals enhance perceptual processing through re-entrant modulation of visual areas (Keil et al. 2009, Sabatinelli et al. 2013). Given MEG’s limited sensitivity to subcortical sources, our findings likely reflect the cortical component of this system—specifically, the enhancement of visual representations.

The FF region showed the clearest own-infant advantage and its longitudinal attenuation. In other visual regions, effects were less consistent: the lingual region showed stable effects without longitudinal modulation, whereas effects in the lateral occipital region were observed only in the late LPP. The FF gyrus plays a central role in face perception and has been implicated in encoding baby schema features (Glocker et al. 2009, Luo et al. 2015). It has also been linked to postpartum-related variation in cortical structure, with FF thickness associated with maternal care experiences and postpartum duration (Kim et al. 2010b, 2018). In addition, this region shows increased functional responses to salient infant cues such as crying (Kim et al. 2010b). The pronounced LPP modulation in this region may therefore reflect its particular sensitivity to infantile facial features and to the subjective salience of one’s own infant. Mothers in our study rated their own infant as more positively valenced than unfamiliar faces, and although these ratings did not correlate directly with neural responses, the combination of higher valence and enhanced FF activity suggests a positive affective bias toward the own infant.

There is also evidence linking FF activity to maternal attachment. Bartels and Zeki (2004) reported that viewing one’s own child activated the FF gyrus, a region not engaged when adults viewed images of their romantic partners. They proposed that FF engagement in this context may reflect the need to continuously update facial representations to interpret a child’s expressions. In this light, our results may reflect a neural mechanism attuned to the specific demands of early parenthood, wherein the FF gyrus plays a central role in processing individualized, emotionally significant infant cues.

### Limitations

Several limitations should be considered when interpreting the present findings. First, infant age at study entry varied across participants (approximately 5–9 months postpartum). Although all mothers were followed over a similar interval and therefore gained a comparable amount of additional caregiving experience, this variability makes it difficult to fully separate the effects of increasing caregiving experience from broader changes associated with progression through the postpartum period. At the same time, the two assessments primarily sampled different phases of early motherhood, with the first assessment centered on the first postpartum year (median infant age = 7.1 months) and the second occurring predominantly after the infant’s first birthday (median infant age = 13.3 months). Exploratory analyses found no evidence that infant age systematically moderated the observed neural response patterns, although the sample size was not well powered to detect small age-related effects. Future studies beginning earlier after childbirth and including more frequent assessments will be important for characterizing the developmental trajectory of neural responses to one’s own infant.

Second, the same photograph of each participant’s infant was presented at both measurement sessions. Using the same stimulus is important for avoiding confounding effects related to differences in the physical characteristics of the stimulus. However, this meant that the photograph did not perfectly match the infant’s current appearance during the second assessment. As discussed above, this may have affected the attentional or motivational significance of the stimulus and contributed to the observed LPP attenuation. At the same time, extensive familiarity with one’s own infant is likely to support a robust identity representation that generalizes across changes in appearance, as has been demonstrated more broadly for personally familiar faces (Burton *et al*. 2005, Burton *et al*. 2011). However, the present design cannot establish whether such a representation retains the same attentional or motivational significance when viewing an earlier photograph. Mothers continued to rate their own infant face more positively than unfamiliar infant and adult faces at both assessments, and neither valence nor clarity ratings changed significantly over time. However, these explicit ratings cannot determine whether the earlier photograph retained the same attentional or motivational significance as a contemporaneous image. In addition, the consistently high positivity ratings for own-infant faces leave relatively little room for detecting a decrease over time. Future studies should directly compare responses to contemporaneous and earlier photographs of the same child.

Third, only neutral facial expressions were used. This choice maximized comparability across stimulus categories and measurement sessions. However, the present findings cannot determine whether similar longitudinal changes would be observed for emotionally expressive infant cues, which may engage affective and motivational systems differently. It is also possible that emotionally expressive infant faces would elicit effects in earlier stages of face processing. In addition, neutral infant faces may not be interpreted similarly by all mothers. Maternal anxiety has been associated with enhanced neural responses to neutral infant faces, possibly because ambiguous infant cues may acquire greater emotional salience for more anxious mothers (Malak *et al*. 2015). Consequently, individual differences in affective state may have contributed to variability in neural responses. Finally, given the modest sample size typical of MEG research, smaller effects in early perceptual or affective processing stages may have remained undetected.

## Conclusions

In summary, this study shows that neural responses associated with motivated attention to one’s own infant attenuate during the transition from infancy to early toddlerhood, whereas responses to unfamiliar infant and adult faces remain largely stable. This attenuation emerged in the LPP time window, suggesting reduced attentional engagement as familiarity and caregiving experience accumulate. In contrast, early perceptual (M170) and early affective valuation processes (OFC activity) remained stable and did not differentiate between face types, indicating that later attentional mechanisms are more sensitive to caregiving experience than early perceptual or affective responses. Together, these findings provide longitudinal evidence that neural sensitivity to one’s own infant is dynamic and changes across early parenthood, consistent with experience-dependent changes in motivated attention to one’s own infant.

## Supplementary Material

nsag073_Supplementary_Data

## Data Availability

The data underlying this article are available in the Open Science Framework (OSF) at https://osf.io/tc4ey/ and a preprint version of this manuscript is available at https://doi.org/10.31234/osf.io/3uhne_v3. The submitted version is identical to the preprint.
