# Supplementary material for: Selective attenuation of neural responses to own infant’s face in early motherhood: a longitudinal magnetoencephalography study
Source: Soc Cogn Affect Neurosci. 2026 Sep 2;21(1):nsag073. doi: 10.1093/scan/nsag073 (PMC13597114; doi:10.1093/scan/nsag073)
Supplement: Supplementary file 1 [file nsag073_supplementary_data.docx]

Supplementary Materials for *Selective attenuation of neural responses to own infant's face in early motherhood: A longitudinal MEG study*

**Participant recruitment, characteristics and questionnaire measures**

Addresses of mothers living in the Jyväskylä region in Central Finland who had recently given birth were obtained from the Finnish Digital and Population Data Services Agency. Recruitment letters were sent to 309 mothers without other children in the household to target first-time mothers. Out of these, 30 mothers volunteered for the study. Six participants were excluded for not meeting the inclusion criteria. Altogether 24 participants were recruited for the study. Three of them cancelled participation before the first measurement. For one participant the MEG measurement could not be conducted due to excessive electrical noise in the initial signal check. Twenty participants completed the first MEG session, and sixteen returned for the follow-up session approximately six months later.

Exclusion criteria included neurological disorders (with the exception of attention deficit disorder, reading disorder, and migraine), currently active psychiatric disorder within the past two years, diagnosis of psychotic disorder, use of medication affecting the central nervous system, belonging to a COVID-19 risk group at the time of data collection, metallic implants incompatible with MEG/MRI, hearing impairment, substance abuse, or other medical or motor conditions preventing participation. Participant eligibility was confirmed during a phone interview based on participants’ self-report.

Background demographic information was collected (e.g., education, employment, household composition). Prior to each MEG session, participants completed online self-report questionnaires assessing depressive symptoms, trait anxiety, and maternal attachment. Depressive symptoms were measured using the 10-item Finnish version of the Center for Epidemiologic Studies Depression Scale (CES-D; Radloff, 1977; Andersen et al., 1994; score range 0–30). Trait anxiety was assessed with the Trait version of the State–Trait Anxiety Inventory (STAI-T; Spielberger et al., 1983; score range 20–80). Maternal feelings of attachment towards the infant were measured with the Maternal Postnatal Attachment Scale (MPAS; Condon & Corkindale, 1998; score range 19–95). Descriptive statistics for these measures are presented in Table S1.

**Table S1**

*Descriptive statistics for questionnaire measures across sessions*

| **Measure** | **First session (M, SD, Range)** | **Second session (M, SD, Range)** | ***t*** | **df** | ***p*** |
| --- | --- | --- | --- | --- | --- |
| CES-D | 6.4 (4.2), 1–15 | 6.9 (4.9), 1–19 | 0.7 | 15 | .243 |
| STAI-T | 35.3 (9.4), 23-56 | 38.0 (9.4), 28–55 | 2.1 | 15 | .051 |
| MPAS | 79.7 (10.8), 50.9–93.6 | 81.2 (8.6), 60.6–93.6 | 2.1 | 15 | .057 |

Note. Values represent means (M), standard deviations (SD), and observed score ranges. T-statistics reflect two-tailed paired t-tests comparing scores between sessions. CES-D = Center for Epidemiologic Studies Depression Scale; STAI-T = Trait version of the State–Trait Anxiety Inventory; MPAS = Maternal Postnatal Attachment Scale.

**Stimuli**

**Image preprocessing**

All face images were preprocessed using GIMP (GNU Image Manipulation Program). To equalize overall brightness across stimuli, mean gray-level intensity was adjusted using GIMP’s Levels tool. Luminance matching was verified using the *lum_calc* MATLAB function from the SHINE_color toolbox (Dal Ben, 2023), which computes mean pixel intensity for each image category on a unitless grayscale scale (0–255). The resulting mean luminance values were highly similar across categories: adult faces (M = 184.2, SD = 0.6), own-infant faces (M = 183.9, SD = 2.1), and unfamiliar-infant faces (M = 184.5, SD = 0.3).

**Stimulus characteristics**

For unfamiliar and adult face stimuli, only identities previously validated as displaying neutral facial expressions were included. Neutral-expression agreement exceeded 73% for NimStim (Tottenham et al., 2009) and mean hit rates were above 85% for KDEF (Goeleven et al., 2007). For the unfamiliar-infant category, TIF images rated as neutral in previous validation work were used, with reported neutral-expression agreement ranging from 52% to 91% (Maack et al., 2017). The unfamiliar infant pool comprised 14 identities (11 from TIF and 3 participant-provided), and the adult face pool comprised 15 identities (7 from NimStim and 8 from KDEF).

**Participant ratings**

After the face task, participants rated the three stimulus images presented during the experiment. Each image was shown once in random order. Participants categorized the facial expression (happy, sad, surprised, disgusted, fearful, angry, or neutral), rated the clarity of the expression 1–5 (1 = very unclear, 5 = very clear), and evaluated the valence of the expression 1–5 (1 = very negative, 5 = very positive). Expression categorization frequencies are shown in Table S2.

**Table S2**

*Participants’ categorization of facial expressions in the stimulus images*

| **Stimulus** | **Neutral** | **Sad** | **Surprise** | **Angry** | **Happy** | **Fearful** | **Disgusted** |
| --- | --- | --- | --- | --- | --- | --- | --- |
| **First session** | | | | | | | |
| Own infant | 10 | 0 | 4 | 0 | 2 | 0 | 0 |
| Unfamiliar infant | 9 | 3 | 1 | 1 | 0 | 2 | 0 |
| Adult | 13 | 1 | 1 | 1 | 0 | 0 | 0 |
| **Second session** | | | | | | | |
| Own infant | 6 | 0 | 7 | 0 | 3 | 0 | 0 |
| Unfamiliar infant | 6 | 2 | 3 | 1 | 0 | 3 | 1 |
| Adult | 12 | 0 | 2 | 2 | 0 | 0 | 0 |

*Note. Values represent the frequency of participants classifying each stimulus image into the indicated expression category. Because each stimulus image was unique to a given participant and evaluated by only one participant, these frequencies describe how the stimuli were categorized in the present sample rather than providing normative validation of the facial expressions.*

At the first session, valence ratings were more positive for the own-infant face (M = 4.0, SD = 0.73) than for unfamiliar infant (M = 3.1, SD = 0.93), *t*(15) = 2.9, *p* = .011, and adult faces (M = 2.9, SD = 0.81), *t*(15) = 4.1, *p* < .001. Clarity ratings were higher for own-infant (M = 4.0, SD = 0.82) than unfamiliar infant faces (M = 3.1, SD = 1.1), *t*(15) = 3.2, *p* = .006, and higher for adult than unfamiliar infant faces, *t*(15) = 3.1, *p* = .008. Valence ratings did not differ between unfamiliar infant and adult faces, and clarity ratings did not differ between own-infant and adult faces.

A similar pattern was observed at the second session. Valence ratings were more positive for own-infant faces (M = 4.1, SD = 0.81) than unfamiliar infant (M = 3.3, SD = 0.77), *t*(15) = 3.1, *p* = .008, and adult faces (M = 2.8, SD = 0.75), *p* < .001. Clarity ratings were higher for own-infant (M = 4.0, SD = 0.97) than unfamiliar infant faces (M = 3.1, SD = 0.96), *t*(15) = 2.8, *p* = .014, and did not differ between own-infant and adult faces (adult: M = 3.7, SD = 0.94) or between unfamiliar infant and adult faces. When comparing the ratings for the same Face type between sessions, neither valence nor clarity ratings differed significantly between sessions.

To examine whether emotion categorizations changed over time, responses were collapsed into neutral versus emotional categories because the frequencies for individual emotion categories were low. Changes in categorizations between sessions were then examined separately for each face type using McNemar tests. No significant changes in categorization were observed for own-infant faces, χ²(1) = 1.50, *p* = .221, unfamiliar infant faces, χ²(1) = 0.80, *p* = .371, or adult faces, χ²(1) = 0.00, *p* = 1.000. Although descriptively own-infant faces were more frequently categorized as emotional at the second session (10/16 participants) than at the first session (6/16 participants), this trend was not statistically significant.

**Procedure**

Upon arrival at the laboratory, participants were prepared for MEG recording. Hearing thresholds were measured using an audiometer for a separate auditory experiment not reported here. Resting-state activity was recorded for 2 minutes with eyes open and 2 minutes with eyes closed at the beginning and end of the session. Participants then completed the face task described in the main text, followed by an auditory task involving infant and adult vocalizations (not reported here). Structural MRI was obtained separately at a private clinic (Synlab Jyväskylä), on average 5.2 weeks after the first MEG session.

**MEG data acquisition**

Five head position indicator (HPI) coils were attached to the scalp (three on the forehead and one behind each ear) and digitized using an Isotrak 3D digitizer (Polhemus, USA), along with three anatomical landmarks (nasion and left and right preauricular points). In addition, more than 100 head shape points were digitized to improve co-registration with magnetic resonance imaging (MRI).

T1- and T2-weighted images were acquired. For T1-weighted images, the acquisition parameters were: TR/TE = 8.36/3.15 ms, flip angle = 12°, matrix size = 256 × 256, slice thickness = 1.2 mm, sagittal orientation. For T2-weighted images, the parameters were: TR/TE = 9684.58/101.45 ms, flip angle = 160°, matrix size = 512 × 512, slice thickness = 3.0 mm, transverse orientation.

The electrooculogram were recorded with bipolar electrodes attached to the outer canthi of each eye (horizontal eye movements) and with one electrode on top of the right eyebrow and one below the right eye (vertical eye movements). One ground electrode was attached to the back of the neck approximately on the T1 thoracic vertebrae. The electrocardiogram was recorded with one electrode attached under the right collarbone and another electrode to left side between the 11^th^ and 12^th^ rib.

During the recording the participant was seated in a dimly lit room to a chair with a 68-degree upright gantry position and the head inside the MEG sensor helmet.

**Test–retest reliability of MEG responses**

To illustrate the consistency of neural responses to the same face type across sessions, significant correlations between the first and second measurement sessions are shown in Figure S1 for components where significant correlations were found.

**Figure S1**. *Test–retest reliability of MEG responses across sessions. Scatter plots illustrate correlations between responses to the same face type across the first and second sessions for M170 (fusiform cortex), early LPP, and late LPP in the lateral occipital cortex (lOCC). Correlation coefficients (r) are shown with asterisks indicating significance levels (*p < .05, **p < .01, ***p < .001).*

**ALT TEXT:** *Scatter plots showing correlations between first and second session MEG responses. Panel A shows positive correlations for M170 across own infant, unfamiliar infant, and adult faces. Panel B shows positive correlations for early and late LPP in the lateral occipital cortex for adult faces.*

**Table S3. Exploratory mixed-effects analyses examining whether longitudinal changes varied as a function of infant age**

| **Outcome measure** | **Session × Infant Age F(df1, df2)** | **p** | **Face Type × Session × Infant Age F(df1, df2)** | **p** |
| --- | --- | --- | --- | --- |
| M170 | F(1, 16) = 0.01 | 0.908 | F(1, 16) = 2.16 | .674 |
| OFC | F(1, 16) = 1.80 | 0.454 | F(1, 16) = 1.31 | .360 |
| Early LPP FF | F(1, 16) = 0.14 | 0.829 | F(1, 16) = 0.25 | .816 |
| Early LPP lOCC | F(1, 16) = 0.95 | 0.540 | F(1, 16) = 0.20 | .824 |
| Early LPP ling | F(1, 16) = 0.01 | 0.985 | F(1, 16) = 0.04 | .962 |
| Late LPP FF | F(1, 16) = 0.75 | 0.874 | F(1, 16) = 1.39 | .314 |
| Late LPP lOCC | F(1, 16) = 0.00 | 0.163 | F(1, 16) = 0.04 | .985 |
| Late LPP Ling | F(1, 16) = 1.74 | 0.734 | F(1, 16) = 0.72 | .439 |

**Note.** Exploratory linear mixed-effects models included Face Type, Session, Infant Age at Session 1, and all interactions as fixed effects, with participant as a random intercept. Infant age at Session 1 was included as a time-invariant covariate.

**References:**

Andersen EM, Malmgren JA, Carter WB, et al. Screening for depression in well older adults: Evaluation of a short form of the CES‑D. *Am J Prev Med* 1994;10:77–84.

Condon JT, Corkindale CJ. The assessment of parent‑to‑infant attachment: Development of a self‑report questionnaire instrument. *J Reprod Infant Psychol* 1998;16:57–76. https://doi.org/10.1080/02646839808404558

Dal Ben R. SHINE_color: Controlling low‑level properties of colorful images. *MethodsX* 2023;11:102377. https://doi.org/10.1016/j.mex.2023.102377

Goeleven E, De Raedt R, Leyman L, et al. The Karolinska directed emotional faces: A validation study. *Cogn Emot* 2007;22:1094–1118. https://doi.org/10.1080/02699930701626582

Maack JK, Bohne A, Nordahl D, et al. The Tromso Infant Faces database (TIF): development, validation and application to assess parenting experience on clarity and intensity ratings. *Front Psychol* 2017;8:409. https://doi.org/10.3389/fpsyg.2017.00409

Radloff LS. The CES‑D Scale: A self‑report depression scale for research in the general population. *Appl Psychol Meas* 1977;1:385–401. https://psycnet.apa.org/doi/10.1177/014662167700100306

Spielberger CD. *State‑Trait Anxiety Inventory for Adults: Sampler Set*. Palo Alto, CA: Mind Garden; 1983.

Tottenham N, Tanaka J, Leon AC, et al. The NimStim set of facial expressions: judgments from untrained research participants. *Psychiatry Res* 2009;168:242–249. https://doi.org/10.1016/j.psychres.2008.05.006
